# Nodulation and nitrogen fixation in *Medicago truncatula* strongly alters the abundance of its root microbiota and subtly affects its structure

**DOI:** 10.1111/1462-2920.16164

**Published:** 2022-08-31

**Authors:** Andrzej Tkacz, Raphael Ledermann, Anna Martyn, Sebastian Schornack, Giles E. D. Oldroyd, Philip S. Poole

**Affiliations:** ^1^ Department of Biology University of Oxford Oxford UK; ^2^ Sainsbury Laboratory University of Cambridge Cambridge UK

## Abstract

The plant common symbiosis signalling (SYM) pathway has shared function between interactions with rhizobia and arbuscular mycorrhizal fungi, the two most important symbiotic interactions between plants and microorganisms that are crucial in plant and agricultural yields. Here, we determine the role of the plant SYM pathway in the structure and abundance of the microbiota in the model legume *Medicago truncatula* and whether this is controlled by the nitrogen or phosphorus status of the plant. We show that SYM mutants (*dmi3*) differ substantially from the wild type (WT) in the absolute abundance of the root microbiota, especially under nitrogen limitation. Changes in the structure of the microbiota were less pronounced and depended on both plant genotype and nutrient status. Thus, the SYM pathway has a major impact on microbial abundance in *M*. *truncatula* and also subtly alters the composition of the microbiota.

## INTRODUCTION

Plants recruit a root‐associated microbial community (microbiota) to specific root niches including the rhizosphere (region within a few mm of the root), the rhizoplane (root surface) and the endosphere (microbiota resident inside roots) (Tkacz et al., 2020). The root microbiota is critical for plant productivity as it is essential for nutrient uptake (Tkacz & Poole, 2015) and primes plant defence responses to promote control of pathogens through the production of antimicrobial metabolites (Berendsen et al., 2012; Bulgarelli et al., 2013). It is a two‐way dialogue, with the microbiota altering plant growth and plants manipulating the microbiota in and around their roots. Plants may exude up to 20% of fixed carbon via their roots, such as small organic molecules and signalling factors (Badri & Vivanco, 2009). Carbon export on this scale is expected to be the major way plants modulate the structure and function of their root microbiota and thus offer significant fitness benefits and promote evolution of mutualisms between plants and microbes (Poole et al., 2018). Evidence that the microbiota is systematically ‘farmed’ by plants comes from studies showing that different species of plants grown in the same soil have distinctive root microbiotas (Tkacz et al., 2020; Wippel et al., 2021).

Two of the best‐characterized mutualisms, are those between rhizobia and legumes leading to root nodulation and plant infection by arbuscular mycorrhizae fungi (AMF). N_2_ fixation by rhizobia in association with crop legumes provides up to 22 Tg of nitrogen a year (Peoples et al., 2009). Around 80% of land plants are infected by AMF which make a substantial contribution to plant nutrition, particularly uptake of phosphate, with rice receiving 70% of their phosphate through this symbiosis (Yang et al., 2012), but also nitrogen and water, among others (Berruti et al., 2016). These crucial symbioses share a common symbiosis signalling pathway (SYM) with nodulation evolving ~100 million years ago (MYA) by adaption of the more ancient AM symbiosis (~ 400 MYA) (Griesmann et al., 2018; van Velzen et al., 2018).

In the model legume of *Medicago truncatula*, nodules are initiated by release of plant flavonoids, which induce *nod* genes in rhizobia encoding enzymes for synthesis of lipo‐chitooligosaccharide Nod factors. *M*. *truncatula* detects Nod factors with LysM‐RLK receptors such as NFP and LYK3 (Amor et al., 2003; Moling et al., 2014). These receptors then signal via the common SYM pathway with a key branch point located after DMI3 (calcium calmodulin‐dependent kinase; CCamK) (Wais et al., 2000) where it splits into nodule‐ and mycorrhiza‐specific paths. The gibberellic acid‐insensitive type regulators NSP1 and NSP2 induce the early nodulin genes (Kaló et al., 2005; Smit et al., 2005). Concomitant with activation of the signalling pathway, rhizobia attached to root hairs are entrapped by their curling, resulting in the formation of an infection chamber. In WT plants, the bacteria enter a plant‐derived infection thread, down which they grow until finally engulfed by plant cells (Poole et al., 2018). However, plants mutated in a gene called *api* (altered primordium invasion) (Gavrin et al., 2020) are blocked in this process just prior to nodule primordium infection and hence develop defective infections (Inf^−^). With time some of these nodules become functional, however, in our 6‐week assays we only observed small white nodules. In WT plants, bacteria differentiate into N_2_‐fixing bacteroids and are provided with dicarboxylic acids as a carbon source in exchange for secretion of ammonia (Udvardi & Poole, 2013). Development of bacteroids leading to N_2_ fixation is controlled by many plant factors, but among the best characterized in *M*. *truncatula* are the antimicrobial nodule‐specific cysteine‐rich (NCR) peptides that control bacteroid development. Plant lines unable to export NCR peptides (*dnf1*) form white nodules containing bacteria blocked in the differentiation process (Inf^−^) (Wang et al., 2010). The mycorrhiza‐specific branch of the SYM pathway after DMI3 (CCamK) includes two key genes, *ram1* and *ram2*, whose mutation prevents mycorrhizal infection of the plant. RAM1 is a transcription factor that induces lipid biosynthesis in the plant cells accommodating fungal arbuscules (Gobbato et al., 2012), while RAM2, a direct target of RAM1, is a glycerol‐3‐phosphate acyltransferase, an essential step in lipid synthesis (Wang et al., 2012). As AMF are fatty acid auxotrophs, plant export of these compounds is critical to AMF survival (Luginbuehl et al., 2017).

Despite an increasing number of studies looking at the microbiota of legumes, no clear conclusions have been drawn regarding the impact of the soil and root microbiota on the potential for colonization by symbionts. Studies looking at *Trifolium* and *Medicago* showed that their roots are covered with the symbiont as 70% and 60% of the total root microbiota consists of *Rhizobium* and *Sinorhizobium* (*Ensifer*), respectively (Brown et al., 2020; Hartman et al., 2017). While other studies reported contrasting much lower values of ~10% for *Mesorhizobium* abundance on *Lotus* roots (Zgadzaj et al., 2016), between 10% and 15% for *Rhizobium* colonization of pea plants roots (Cordero et al., 2020; Horner et al., 2019), approximately 1% of *Bradyrhizobium* in pigeon pea plants (Chalasani et al., 2021) and below 10% for the *Ensifer* root load in *Medicago* plants (Tkacz et al., 2020). Whether these discrepancies are due to different sampling strategies and/or growth conditions needs to be established. Moreover, SYM mutants of *Lotus japonicus* do not have significantly reduced root colonization by their *Mesorhizobium* symbionts, suggesting plants have an SYM‐independent way of attracting compatible symbionts (Zgadzaj et al., 2016). However, this study reported that plant SYM mutants significantly alter the structure of the rhizosphere and root microbiota compared to WT plants.

There is a lack of quantitative information for plant microbiota as most metagenomic studies rely on PCR‐based assays where only relative abundances are captured. However, with the development of quantitative methods, such as flow cytometry, the importance of absolute quantitation in metagenomic studies was revealed from the study of gut microbiota and development of Crohn's disease (Vandeputte et al., 2017). Plant‐associated microbiota studies have utilized our previously introduced spike‐in system (Tkacz et al., 2018). For example, Wang et al. (2020) showed that, in general, the plant rhizosphere is an order of magnitude better colonized by bacteria than the surrounding soil. Moreover, an increase in the total abundance of bacteria on roots was shown during drought stress in rice and fungal root rot infection in wheat (Guo et al., 2020). The mechanistic explanation for these observations is not clear, although an increase in microbial abundance may result from elevated secretion by plant roots. However, an increase in microbial abundance may also be associated with a reduced immune response, as occurred under nutrient limitation (Castrillo et al., 2017).

Here, we show that the plant‐nutritional status is a key determinant of the abundance of the microbiota. Plants grown under limiting‐N and unable to obtain it through symbiosis had a significantly lower number of prokaryotes compared to non‐starved plants. While we detected a statistically significant change in the microbiota structure between WT and SYM mutants this was less drastic than the effect of either the fraction (rhizosphere v root) or the sampling time (6 vs. 12 weeks).

## EXPERIMENTAL PROCEDURES

### Plant growth and sampling

For all experiments, *Medicago truncatula* A17 was used as WT (Nod^+^ and Myc^+^, i.e., able to nodulate and mycorrhizae). *M*. *truncatula* mutant lines, *dmi3* (Nod^−^ and Myc^−^), *nfp1* and *nsp1* (Nod^−^ and Myc^+^), *ram1* and *ram2* (Nod^+^ and Myc^−^), *api* and *dnf1* (Inf^−^ and Myc^+^) are all derived from A17 (Amor et al., 2003; Breakspear et al., 2014; Gavrin et al., 2020; Gobbato et al., 2012; Teillet et al., 2008; Wais et al., 2000; Wang et al., 2010). Ten plant biological replicates were used per condition, apart from *ram2* grown in complete +N + P condition (*n* = 4) and hence, growth of this plant was compared only against *ram1*. For experiments where plants were inoculated with their native symbiont; 10^5^ cells of *Ensifer* (formerly *Sinorhizobium*) *medicae* strain WSM419 were added.

Plant seeds were surface sterilized using bleach (4%) and ethanol (70%) and germinated on MS (Murashige & Skoog, 1962) agar plates. Seedlings were placed in 100 ml pots filled with farm soil or 1 part soil /9 parts silver sand mixture (10% soil) and grown in a glasshouse under for 6 weeks (in one experiment plants were also harvested after 12 weeks). Two types of farm soil were used; nutrient‐rich Wytham and nutrient‐poor Bawburgh (as used previously, Tkacz et al., 2020), as well as 10% Wytham soil. The rhizosphere was sampled as a soil fraction adhering to roots after manual shaking and subsequently vortexed from roots at 2000 rpm for 5 min using Multi Reax shaker (Heidolph, Schwabach, Germany). The root fraction was obtained by macerating the root tissue using mechanical lysis with bead tubes. Hence, we consider tightly attached microbes as well as root endophytes as a part of our root fraction samples. For the *Medicago* genotypes able to nodulate, any visible nodules were removed before sampling. For all experiments, unplanted potted soil controls were kept alongside planted pots in the greenhouse. Soil in these pots was mixed and sampled to give ‘bulk soil’ fraction. Where root tips were sampled, the terminal 2 cm of each lateral and primary root were removed. Plant shoots were air‐dried at 60°C for 7 days and weighted.

Soil chemistry was determined by Hutton‐Analytical (Aberdeen). Bawburgh soil (pH 7.5) contained P^−3^ 120.5 mg kg^−1^, K^+^ 168 mg kg^−1^, Mg^2+^ 33.6 mg kg^−1^ and 2.92% organic matter. Wytham soil (pH 7.2) contained P^−3^ 122.9 mg kg^−1^, K^+^ 483 mg kg^−1^, Mg^2+^ 304 mg kg^−1^ and 16.8% organic matter. For experiments with diluted (10%) soil, rooting solution (20 ml per plant) containing KH_2_PO_4_ (25 g/L) and Na_2_HPO_4_ (28.4 g/L) and KNO_3_^−^ (500 mg/L) was added (Poole et al., 1994). Depending on the desired condition, the solution was modified to exclude either the phosphorous (+N − P condition) or nitrogen (−N + P condition). We have previously established that addition of 10 mg of KNO_3_ per plant (20 ml of 500 mg/L stock) allows nodulation of *Medicago* plants by native or added symbionts, but prevents nodulation mutants from yellowing.

### DNA isolation and spike addition

Soil and root samples (300 mg) were placed in ZR Soil Microbe DNA Kit bead tubes (D6010, Zymo Research, Irvine, CA, USA). Microbial DNA was released using three rounds of bead beating at 10 m s speed for 30 s^−1^ (FastPrep‐24 5G Grinder, MP Biomedicals, Santa Ana, CA, USA). Further DNA isolation and purification were conducted according to D6010 kit specifications. Isolated DNA was quantified using Qubit BR dsDNA and standardized to 5 ng/μl concentration for subsequent PCR.

To quantify the number of prokaryotic 16S rRNA gene copies, we added prokaryotic P‐spike plasmids as previously (Tkacz et al., 2018). Briefly, the plasmid contains a single copy of a synthetic 16S rRNA gene mimic with primer binding sites for the prokaryotic 515F and 806R primers widely used in soil microbiology (Caporaso et al., 2011). Between these primer binding sites, there are 253 bp of synthetic DNA, the sequence of which is not found in any known living organism (Tkacz et al., 2018). P‐spike plasmid was co‐isolated along with microbial DNA, co‐amplified during PCR and sequenced along with microbial DNA‐derived amplicons. The microbial 16S rRNA gene copy number was calculated based on the sequencing output using the number of reads annotated as microbial and synthetic (matching our synthetic DNA sequence) and the amount of P‐spike added into the samples prior to DNA isolation.

$$\begin{array}{l} \frac{P-\text{spike plasmid copies added(known)}}{\text{microbial}\;16S\;\text{rRNA gene copy in soil or root}}\\ \;=\frac{\text{synthetic reads(known after sequencing)}}{\text{microbial reads(known after sequencing. )}} \end{array}$$

DNA spikes (3 μl) were added to each DNA isolation tube. For the bulk soil samples, the DNA spike concentration was 0.15 pg/μl (or 0.45 pg in total), based on Qubit BR dsDNA (Invitrogen, Carlsbad, CA, USA) measurement. Previously, we established the quantity range for P‐spike addition for soil samples. However, as the spiking method has not been used for root samples, we diluted the P‐spike concentration 10‐fold due to lower prokaryotic abundance in this environment (Wang et al., 2020). To avoid DNA spike degradation, immediately after spiking the lysis solution was added (D6001, ZYMO Research) and microbial DNA was released into the solution with three rounds of bead beating as already described.

### PCR amplification and sequencing

For the soil and root microbiota assay, protein nucleic acid PCR clamps (1 μl) targeting plastids and mitochondria (PNA Bio, Newbury Park, CA, USA) were added as published (Lundberg et al., 2013) and the 16S rRNA gene was amplified using barcoded 515F and 806R primers (Caporaso et al., 2011). The components of PCR mixture were as follows: Phusion High‐Fidelity (0.2 μl), HF buffer (4 μl) (F520L, Thermo Scientific, Waltham, MA, USA), dNTPs (0.4 μl), primers (1 μl of 10 μM each), template DNA (1 μl of 5 ng/μL) and H_2_O to 20 μl. PCR cycling conditions were as follows: 98°C for 1 min, 30 cycles of: 98°C for 10 s, 55°C for 30 s and 72°C for 45 s, with a final extension at 72°C for 7 min. PCR products were pooled and sequenced using Illumina Miseq 300 bp pair‐end or Novaseq 250 bp pair‐end.

### Data analysis

Paired‐end reads were aligned and filtered by the quality and required sequence length as previously (Tkacz, mBIO 2020). The bioinformatic code is available as Supplementary File S1. For each sample, spike‐origin reads were counted based on their unique sequence, and their number compared against the number of microbial‐origin reads after chimera, mitochondria and chloroplast sequences were removed. Pair‐end reads were merged using usearch10 fastq_mergepairs and quality filtered using usearch10 fastq_maxee set to 1.0. After removal of spikes, mitochondria and chloroplast reads, the remaining microbial reads were clustered into zOTUs using usearch10 with Unoise3 chimera removal (Edgar, 2016) and each zOTU annotated with the SILVA 132 database (Quast et al., 2012). zOTU‐based PCoA and canonical analysis of principal coordinates (CAP) plots, PERMANOVA statistics (type III, unrestricted permutation of raw data with 9999 permutations) were calculated using PRIMER 6 software. Data was statistically assessed and visualized using GraphPad PRISM 6 software. Microbiota abundance was statistically compared using one‐way ANOVA with a Benjamini‐Hochberg correction test for multiple comparisons of means of each condition against every other condition. Plant dry weight and Shannon Diversity Index were statistically compared using one‐way ANOVA with Dunnett's test

## RESULTS

We wanted to decipher the factors controlling assembly of *M*. *truncatula* root and rhizosphere microbiota, including their total abundance, using plants grown on various soil types, nutrients, rhizobial inoculation and sampling times. We were interested in understanding the influence of the plant SYM pathway and other symbiotically important genes as well as nutrient deficiency on the total prokaryotic root microbiota structure and abundance, but especially on bacteria in the genus *Ensifer*, its symbiont.

### Effects of N and P on the plant growth and the root microbiota abundance of *nod*, *myc* and *inf* mutants

**FIGURE 1:**
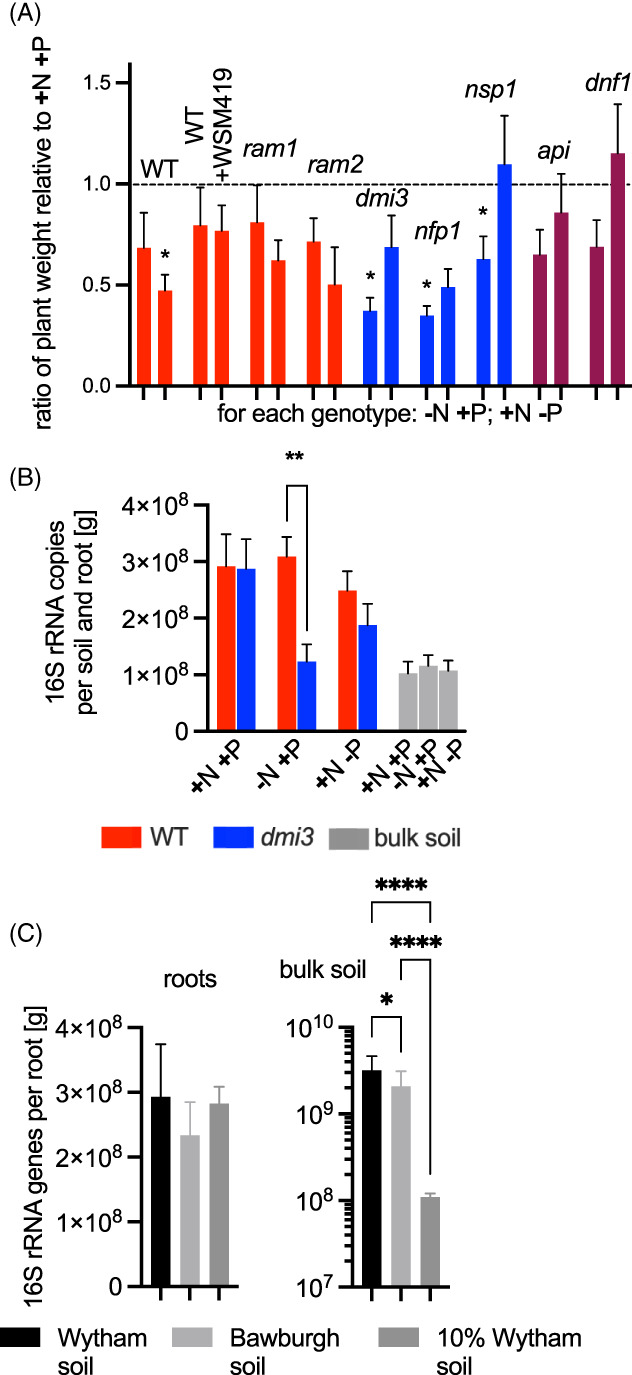
Plant growth and their microbiota absolute abundance. (A) Shoot dry weight of different *M*. *truncatula* genotypes relative to +N + P‐fed plants with the separation for their nodulation phenotype – Nodulation proficient (Nod^+^), nodulation blocked (Nod^−^) or infection impaired (Inf^−^). For each genotype in each nutrition level *n* = 8–10. Error bars represent SE and asterisk indicate a statistical significance using ANOVA with many‐to‐one Dunnett's comparison run against respective +N + P condition (apart from *ram2* being compared against *ram1* plants). Plants were grown in 10% Wytham soil, (B) microbiota abundance of *Medicago* WT and *dmi3* roots and soil under different nutritional conditions in 10% Wytham soil, (C) microbiota abundance of *Medicago* WT grown in Wytham, Bawburgh and 10% Wytham soil (all nutrient level conditions combined) Error bars represent SE and asterisk indicate an outcome of one‐way ANOVA with Benjamini test (for A, C and B – bulk soils) or *t*‐test between WT and *dmi3* for each condition separately. For C ‘roots’; *n* = 4, 4 and 49, respectively, for C ‘bulk soil’; *n* = 9, 7 and 112, respectively, for A ‘roots’; *n* = 40, 8, 36, 12, 36 and 10, respectively and ‘bulk soil’ *n* = 12, 19, 18, respectively.

The effect of nutrient status on plant growth and their microbiota abundance was investigated using different soil types and by changing the levels of N and P (Figure 1). To ensure tight control of nutrient levels, soil was diluted to 10% with sand and defined nutrient solution added. When grown under limiting N, nodulating plants were slightly smaller than N‐fed plants but none of the differences were significant. Thus, as expected the presence of N_2_‐fixing nodules supports the N requirement of plants and prevents starvation. Consistent with this, plants unable to nodulate (*dmi3*, *nfp1 and nsp1*) had significantly reduced growth on limiting exogenously added N. The dry weight of plants with a Inf^−^ phenotype (*api* and *dnf1*) followed the same trend as Nod^−^ plants, although differences were not statistically significant. It should be noted there was no significant effect of inoculation with *Ensifer medicae* strain WSM419, which is a very effective microsymbiont of *M*. *truncatula*, since the WT nodulated well with native rhizobia present in the soil.

Plants grown under limiting P generally had reduced dry weight, with a significant effect for WT (*p* < 0.05) and close to significant for other Nod^+^ genotypes (Figure 1A). Plants unable to form a mycorrhizal symbiosis (*ram1*, *ram2* and *dmi3*) were not obviously disadvantaged by P‐limitation compared to WT. This is consistent with no or limited mycorrhization occurring, even for Myc^+^ plants, in these short‐term growth experiments and explains why the WT had significantly reduced growth in the absence of exogenously added P.

The abundance of root‐associated microbiota was the same for WT and for *dmi3* plants in complete NP medium, that is, absolute prokaryotic abundance does not depend on a functional SYM pathway. However, under N‐limitation but not P‐limitation, the *dmi3* mutant had a significantly reduced prokaryotic abundance per gram of roots. This is consistent with plant growth, with WT forming nodules and fixing N_2_ and relieving N starvation. It also suggests that under the growth conditions used in these studies, lack of N is more limiting to plant growth than lack of P. Of course, the total prokaryotic abundance per plant was greater in WT than in the *dmi3* mutant because WT plants were bigger (Figure S1), however, WT plant roots were also more densely colonized than *dmi3* as shown using prokaryotic 16S rRNA abundance per gram of root (Figure 1B).

The root‐associated microbiota of WT plants grown in 100% soil (Wytham and Bawburgh) or diluted soil (10% Wytham) was consistently ~2–3 x 10^8^ 16S rRNA genes per gram of roots (Figure 1C). This shows the microbiota colonizing the roots assembles independently of the soil nutrient level and microbial abundance in the soil surrounding the roots. There was a small (yet significant) difference in the microbiota abundance between Wytham and Bawburgh bulk soil controls and, as expected, a ~ 10‐fold decrease in the number of prokaryotes in the diluted (10% Wytham) soil (from ~10^9^ to 10^8^ 16S rRNA genes per 1 gram of soil) (Figure 1C). Overall, it suggests that root secretion is the main factor controlling microbiota abundance.

### The structure of the root‐associated microbiota is altered in SYM, cell wall architecture and NCR transport mutants

**FIGURE 2:**
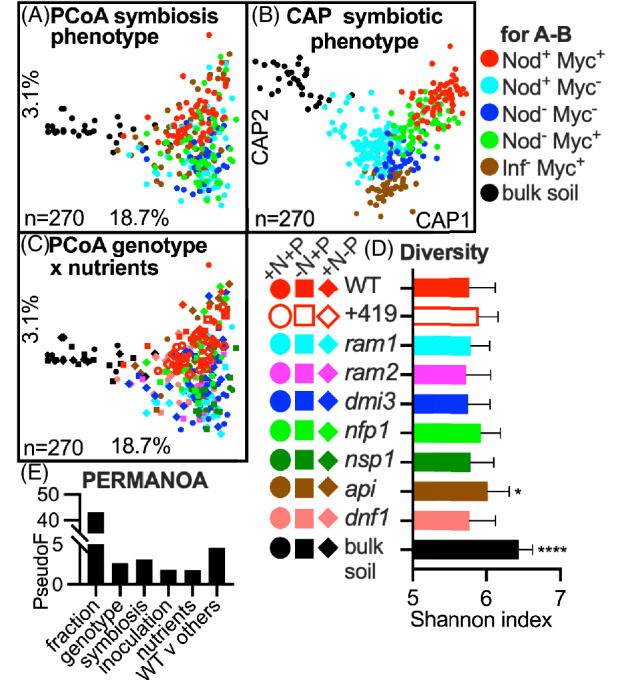
The root microbiota structure is subtly influenced by the plant genotype. Principal coordinate analysis (PCoA) and Canonical analysis of principal coordinates (CAP) plots of microbiota structure visualized focusing on (A) fraction (soil vs. roots) and plant symbiotic phenotype (B) as (A) but shown using CAP using plant symbiotic phenotype as a separation factor, (C) fraction (soil vs. roots) and plant genotypes, (D) Shannon Index showing diversity, with an asterisk indicating a significance using analysis of variance (ANOVA) with many‐to‐one Dunnett's comparison run against WT and (E) permutational multivariate analysis of variance (PERMANOVA) showing a strong influence of fraction and smaller influence of genotype, symbiosis phenotype, inoculation, nutrients and mutation (WT vs. others). +419 means addition of *Ensifer medicae* WSM419, Inf, Infection; Myc, mycorrhization; Nod, nodulation; WT, Wild type

**FIGURE 3:**
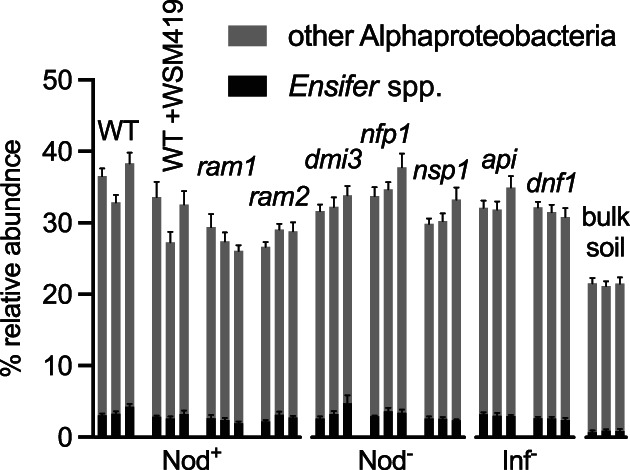
*Ensifer* spp. and other Alphaproteobacteria are attracted to roots independent of the nodulation phenotype of the plant. Percentage of symbiotic genus of *Ensifer* spp. and remaining Alphaproteobacteria in the root‐associated microbiota of *Medicago* WT and other Nod^+^ genotypes, Nod^−^ and Inf^−^ genotypes grown under different nutrient conditions. For each genotype subsequent bars indicate +N + P, −N + P and + N −P growth conditions, *n* = 300

The root microbiota structure of WT and of SYM plants, shared many of its characteristics irrespective of the nutrient conditions during growth (Figure 2). While bulk soil mainly contained Acido‐ and Actinobacteria and to a lesser degree Proteobacteria, plant roots were predominantly colonized by Proteobacteria (Alpha, Beta and Gamma classes) (Figure S2). There was little variation between plant genotypes in the root‐associated taxonomic profile (Figure S2). A detailed analysis of the rhizobial community with a focus on the symbiotic genus of *Ensifer*, showed that along with the Alphaproteobacteria, these groups are enriched in the root‐associated fraction compared to the bulk soil, but enrichment was not dependent on the SYM pathway (Figure 3).

The fraction sampled, whether soil, rhizosphere or root, was the dominant factor shaping the microbiota structure (Figures 2A and S3). All other factors including plant genotype and their symbiotic potential, inoculation with the efficient N_2_ fixing symbiont *E*. *medicae* WSM419, nutrient addition had a subtle, yet statistically significant influence on the composition of the root microbiota (Figures 2A–C,E and S3). A detailed CAP analysis using plant symbiotic phenotype as a separating factor was only partially able to separate these plants from each other (Figure 2B).

The observed diversity of prokaryotes is higher in the bulk soil than in roots (Figure 2D), however, not statistically different between roots of WT and SYM mutants, apart from a difference between WT and *api* genotype (Figures 2D and S3D). Surprisingly, all plant genotypes had an increased diversity of their rhizopshere microbiotas, even though most if not all of the rhizosphere microbial species probably originate from the surrounding bulk soil (Figure S3D). We suspect a reduction in microbiota diversity over time in the pots containing bulk soil causes this effect. Here, the community was exposed to glasshouse, rather than in‐field conditions and was likely to be nutrient starved, which resulted in selection for the most robust species.

There were no significant differences between plant genotypes in root colonization by *Ensifer* for any genotypes examined (after nodules were removed) (Figure 3).

### Spatial and temporal comparison of WT and *dmi3* microbiota

**FIGURE 4:**
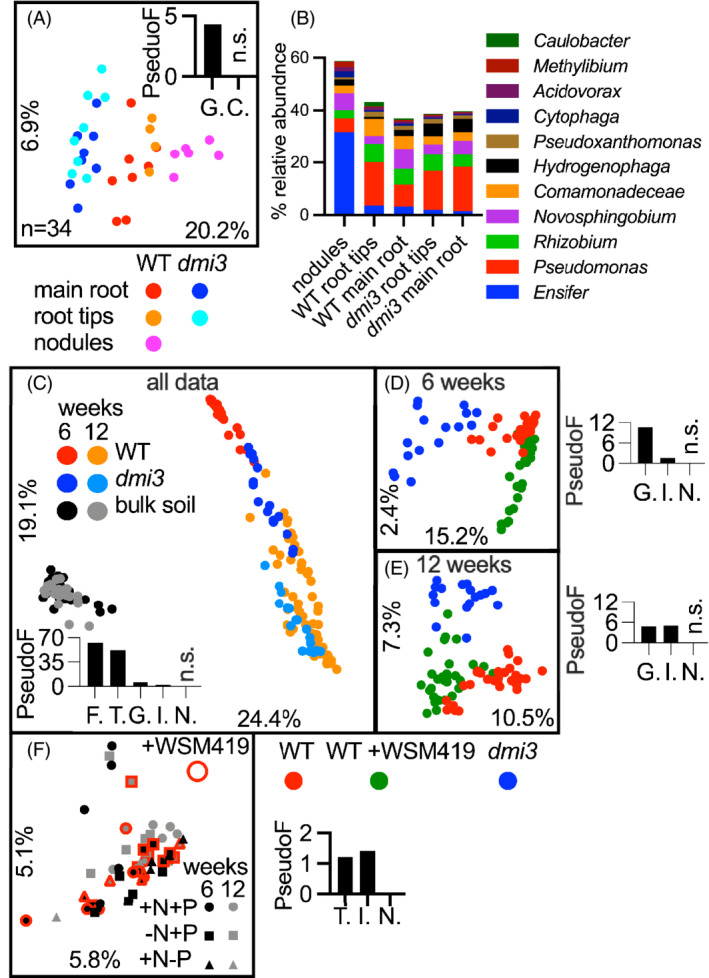
Spatial and temporal comparison of root microbiota of WT v *dmi3*. (A,B) A spatial comparison of WT versus *dmi3* with separation for their root tips and the primary and lateral root (main root), with (A) showing a PCoA plot and PERMANOVA separating plant genotypes (G) but not the tips from the main root (F), and (B) taxonomic assignment at the genus level of nodules and roots of WT and *dmi3* plants, (C–E) PCoA plots and PERMANOVA statistics of WT versus *dmi3* sampled at two different time points; (C) both time points; with WT and WT + WSM419 combined in the plot, (D) 6 weeks only and (E) 12 weeks only, (F) PCoA and PERMANOVA showing bulk soil control samples under different nutrient status and sampling times. Abbreviations for PERMANOVA statistics: C, Compartmentalisation (main root vs. root tips), F, fraction (roots vs. bulk soil), T, time (roots or bulk soil controls at 6 weeks vs. 12 weeks), G, genotype (WT and WT + WSM419 v *dmi3*), I, inoculation with *Ensifer medicae* WSM419 (WT vs. WT + WSM419) and N.: Nutrients (+N + P vs. −N + P v + N −P)

Since the microbiota of WT plants grown for 6 weeks subtly differs from SYM mutants, the microbiota from colonization of different parts of the root and at different plant ages were measured. A large proportion of plant root secretion and bacterial attachment occurs at root tips (Massalha et al., 2017; Pini et al., 2017), hence, tips were separated from the rest of the root in order to test whether there is a difference between plant genotypes in the spatial distribution in recruitment of the microbiota. There was no significant difference in the spatial (main root vs. root tips; i.e., compartmentalisation C) root‐associated microbiota composition both for WT and *dmi3* as shown using PERMANOVA's PseudoF (Figure 4A,B). *Ensifer* as in the other experiment, makes up a minor contribution to both main root and root tip microbiota although it was highly abundant in nodules (Figure 3B). Again, as in the previous experiments, at the whole community level these genotypes (abbreviated as G in Figure 4A) had root microbiota structures which were significantly separated from each other.

Next, plants were sampled after 6 and 12 weeks, to track changes in microbiota assembly dynamics. Apart from the expected changes caused by the fraction (root vs. bulk soil; Fraction: F) there were also significant changes in the structure of the microbiota between plants at 6 and 12 weeks (Figure 4C; Time: T). The sampling time also altered the microbiota of the bulk soil controls with a small, yet significant shift (Figure 4F). However, the time effect was ~40 times stronger for plant roots than for bulk soil (PseudoF for root and bulk soil; 52 and 1.2, respectively) (Figure 4C,F), indicating that the plants developmental age plays a critical role in root microbiota assembly. The effect of genotype on the structure of the community was stronger for 6‐ compared to 12‐week‐old plants (WT v *dmi3*; Genotype: G; PseudoF 10.7 and 4.8, for time points 6 and 12 weeks, respectively; Figure 4D,E), which is consistent with previous DNA‐fingerprinting‐based observations (Mougel et al., 2006), where young plants strongly influenced the structure of their microbiota. The relative strength of factors was conserved across these two sampling time points with genotype being stronger than inoculation: I or nutrient availability: N (Figure 4D,E). The microbiota of bulk soil was relatively stable, yet significantly affected by time and inoculation (Figure 4F).

## DISCUSSION

Under the growth conditions used in these experiments, the absolute abundance of the root‐associated microbiota was governed by the nutritional status of the plants. This was particularly severe for N‐starved plants. An SYM pathway mutant in *dmi3* is unable to fix N_2_ and when grown without added N there was a significantly reduced abundance in the root‐associated microbiota (Figure 1). P had a less dramatic effect on the root microbiota abundance in these experiments, either due to the levels of this nutrient still being sufficient for the plant growth and/or due to low AMF colonization, irrespectively of the nutritional status of the plants. Furthermore, since the abundance of the microbiota on WT plant roots did not change with the nutrient level in the soil (i.e., 100% vs. diluted (10%) soil; Figure 1C), we hypothesise that the root microbiota abundance is primarily dependent on plant exudates.

Crucially, the reduced total microbiota abundance differences did not lead to major changes in the composition of the microbiota (Figure 2). Similarly, wheat plants infected with fungal root rot, which had three times higher bacterial and fungal counts compared to the healthy plants, did not have an altered composition of the root microbiota (Guo et al., 2020). Our absolute quantitation of the root microbiota abundance is comparable with the results obtained in a similar study looking at *Medicago* WT A17 genotype microbiota performed with the same DNA‐spiking system as used here (Wang et al., 2020). In agreement with Wang et al. we also observe a slight increase in the root microbiota abundance compared to bulk soil (i.e., ~3 ×10^8^ and ~1 × 10^8^, respectively) (Wang et al., 2020). N‐starved plants have a reduced microbiota abundance, however, there is a far less dramatic shift in the microbiota structure due to nutrient limitation compared to the root effect (root vs. bulk soil) or plant age (6 weeks vs. 12 weeks) at sampling (Figures 2 and 4). N and P addition do have a subtle, yet significant, effect on the root microbiota structure, an effect also observed in wheat (Pagé et al., 2019). In that study, wheat plants were grown in natural soil with a long history of nutrient amendment and the microbiota differences were observed both for the roots but also for the bulk soil. In our case where soil nutrient differences were not as dramatic, we did not observe changes in the bulk soil alone. A study on *Arabidopsis* found no impact of P‐starvation on the structure of the microbiota; however, plant mutants affected in their P‐starvation response differed in their microbiota structure compared with WT plants (Finkel et al., 2019), indicating that the influence of P on the root microbiota is indirect and mediated by the plant P‐starvation response genes (Castrillo et al., 2017). We have used selected mutants unable to establish a functional symbiosis with AMF (Luginbuehl et al., 2017), which likely prevented the plant from acquiring all the necessary P, however, their root microbiota was in general very similar to the WT (Figure 2).

A multi‐factorial analysis of the structure of the root microbiota of WT and *dmi3* plants confirmed that the differences between these genotypes are small. Moreover, there are no obvious differences in the microbiota structure for different regions of the roots, albeit nodules of WT plants harbour a clearly distinct microbiota dominated by symbiotic *Ensifer* (Figure 4AB). A low abundance of *Ensifer* even on the roots of WT plants (~1%–5%, Figures 3 and 4B) contrasts to the study by Brown et al. where *Medicago* plants, albeit a different genotype from ours, were heavily colonized by this genus with the rhizosphere colonization of ~40%, root of ~60% and nodules of above 80% (Brown et al., 2020). Such differences are unlikely to be caused by the genotypes used, but may reflect different growth conditions and inoculation densities.

The api genotype shows a significant increase in the microbiota alpha‐diversity. This mutant was reported to have impaired oomycete pathogen but also rhizobial symbiosis. The pathogen phenotype is caused by an altered architecture of the plant cell wall (Gavrin et al., 2020) at root tips. Given the fact that many soil bacteria predominantly accumulate at root tips (Massalha et al., 2017), less competitive species may thus find a niche in the roots of this genotype. Seedlings of *api* mutants also secrete less xyloglucans into the soil environment (Gavrin et al., 2020), and reduced xyloglucan secretion may increase soil aggregation, possibly resulting in increased aeration and water movement (Galloway et al., 2018). Hence, in the future, we would like to compare the influence of other plant cell wall‐affected genotypes on their microbiota.

Our results of a small but significant separation of the microbiota of nodulation mutants from that of WT agrees with a study of the *Lotus* microbiota (Zgadzaj et al., 2016), where only a small percentage of the microbiota structural variation was associated with the plant genotype, and it varied due to soil conditions. Moreover, in both studies, the abundance of bacteria from the symbiotic genus on the roots did not differ between WT and SYM‐mutated plants (Zgadzaj et al., 2016). In contrast to the subtle changes observed in the structure of the microbiota, we show that the overall abundance changes drastically in response to the nutritional status of the host plant.

## AUTHOR CONTRIBUTIONS

Andrzej Tkacz and Philip S. Poole conceived and planned the study. Andrzej Tkacz designed the study and Andrzej Tkacz and Anna Martyn performed the experiments. Andrzej Tkacz, Raphael Ledermann, Sebastian Schornack and Giles E. D. Oldroyd and Philip S. Poole analysed the data and drafted the manuscript, and all authors critically revised and approved the final version of the manuscript.

## CONFLICT OF INTEREST

The authors declare that they have no competing interests.

## Supporting information


**Figure S1** Shoot dry weight of different *Medicago truncatula* genotypes separated by their nodulation phenotype—nodulation proficient (Nod^+^), nodulation blocked (Nod^−^) or impaired (Inf^−^). For each genotype in each nutrition level *n* = 8–10, average *n* = 9.3 while *ram2* in +N + P condition was impaired due to technical reasons to I = 4. Error bars represent standard error and * indicate a statistical significance using ANOVA with many‐to‐one Dunnett's comparison run against respective +N + P condition.

**Figure S2** Taxonomic profiles of the rhizosphere and root microbiota of *Medicago* WT and its SYM mutants. *For the rhizosphere, *dnf1* and *api* were not tested. *N* = 137 and 270 for the rhizosphere and root samples, respectively.

**Figure S3** Rhizosphere microbiota structure is influenced by the plant genotype. PCoA plots of microbiota structure visualized focusing on (A) fraction (soil vs. rhizosphere), (B) symbiosis phenotype and (C) plant genotype. (D) Shannon Index showing diversity with an asterisk indicating a significance using ANOVA with many‐to‐one Dunnett's comparison run against WT, (E) PERMANOVA showing a strong influence of fraction and smaller influence of genotype, symbiosis phenotype, inoculation, nutrients and mutation (WT vS. others). +419 means addition of *E*. *medicae* WSM419, Myc, mycorrhization, Nod, nodulation; WT, wild‐type.

**Appendix S1** Supporting information

**Table S1** Supporting information

## Data Availability

Sequencing data for this project are available through the EBI short read archive (primary accession no. PRJEB48524). Additional information about the EBI data submission and the data used to produce the figures can be found in Table S1. The bioinformatic code used to analyse the raw NGS data is provided as Supplementary File S1.
