# Supplementary figures and images for: Nodulation and nitrogen fixation in Medicago truncatula strongly alters the abundance of its root microbiota and subtly affects its structure
Source: Environ Microbiol. 2022 Aug 31;24(11):5524–33. doi: 10.1111/1462-2920.16164 (PMC9804836; doi:10.1111/1462-2920.16164)

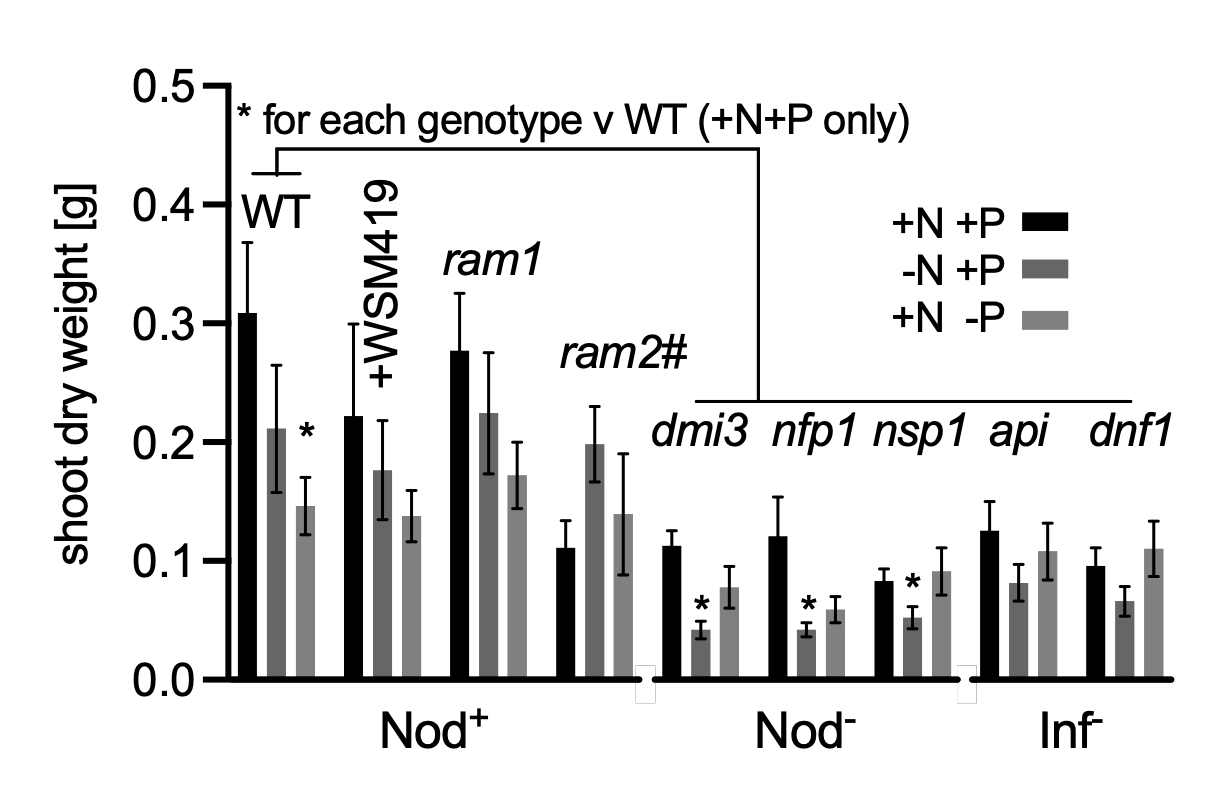

Supplement: Supplementary file 1 — Figure S1 Shoot dry weight of different Medicago truncatula genotypes separated by their nodulation phenotype—nodulation proficient (Nod+), nodulation blocked (Nod−) or impaired (Inf−). For each genotype in each nutrition level n = 8–10, average n = 9.3 while ram2 in +N + P condition was impaired due to technical reasons to I = 4. Error bars represent standard error and * indicate a statistical significance using ANOVA with many‐to‐one Dunnett's comparison run against respective +N + P condition. [file EMI-24-5524-s005.tiff]
